# ERα-mediated cell cycle progression is an important requisite for CDK4/6 inhibitor response in HR+ breast cancer

**DOI:** 10.18632/oncotarget.25552

**Published:** 2018-06-12

**Authors:** Karineh Petrossian, Noriko Kanaya, Chiao Lo, Pei-Yin Hsu, Duc Nguyen, Lixin Yang, Lu Yang, Charles Warden, Xiwei Wu, Raju Pillai, Lauren Bernal, Chiun-Sheng Huang, Laura Kruper, Yuan Yuan, George Somlo, Joanne Mortimer, Shiuan Chen

**Affiliations:** ^1^ Department of Cancer Biology, Beckman Research Institute of the City of Hope, Duarte, CA, United States; ^2^ Department of Breast Health, National Taiwan University Hospital, Taipei City, Taiwan; ^3^ Molecular Pathology Core, Beckman Research Institute of the City of Hope, Duarte, CA, United States; ^4^ Integrative Genomics Core, Beckman Research Institute of the City of Hope, Duarte, CA, United States; ^5^ Department of Surgery, City of Hope Medical Center, Duarte, CA, United States; ^6^ Department of Medical Oncology and Therapeutics Research, City of Hope Medical Center, Duarte, CA, United States

**Keywords:** CDK4/6 inhibitors, palbociclib, patient-derived xenografts (PDX), single cell analysis, DEPArray

## Abstract

While ER has multiple biological effects, ER-cyclin D1-CDK4/6-RB is a critical pathway for the action of estrogen on the cell cycle, especially for breast cancers that rely on estrogen for growth. The latest and most efficient CDK4/6 inhibitors target the phosphorylation of retinoblastoma (RB) tumor suppressor gene; thus, altering levels of many cell cycle molecules. Estrogen receptor (ER)+/HER2- breast cancers have shown great progression free survival when CDK4/6 inhibitors are combined with endocrine therapies. Here we report the mechanism of antiestrogen (fulvestrant) combination with CDK4/6 inhibitors is due to synergism in the suppression of ER-mediated cell cycle progression. Furthermore, we performed single cell analysis of cells from an estrogen dependent/hormone receptor-positive patient derived xenograft (PDX) tumor model treated with palbociclib. These single cells expressed various levels of ER and RB which are involved in cell cycle regulation; and the response to palbociclib treatment relies not only on the ER-cyclin D1-CDK4/6-RB pathway but it is also dependent on elevated levels of ER and/or RB. Our preclinical studies show that palbociclib response is dependent on cells with ER, which is directly involved in cell cycle progression in hormone receptor positive (HR+) breast cancer.

## INTRODUCTION

Breast cancer is the most common cancer in the female population worldwide. Currently, breast cancer is classified into 5 molecular subtypes of which 70% express the estrogen receptor (ER) [1]. The ER has been shown to participate in a number of regulatory mechanisms in hormone receptor positive (HR+) breast cancer [2–4]. Also, ER is known to be involved in cross-talk with alternative growth factor pathways such as PI3K/AKT/mTOR or RAS/RAF/MEK/ERK [5–7]. Moreover, estrogen binding to the ER, promotes the cell cycle progression of HR+ breast cancer cells [8, 9] while ER antagonists (fulvestrant) have been reported to inhibit estrogen’s mitogenic activity on the cell cycle [10–12].

The G1 progression of the cell cycle is controlled by cyclin D1, which is an ER upregulated gene [13, 14], as well as by cyclin E and their catalytic partners: CDKs 4, 6 and 2, respectively. The binding of cyclin D1 to CDK4/6 inactivates retinoblastoma (RB) tumor suppressor, through its phosphorylation, preventing the formation of RB-E2F complex and maintaining the cell cycle progression [15]. Therefore, inhibition of CDK4/6 would block G1 progression. Many cancers display dysregulation of cell cycle networks [16], and the ER-cyclin D1-CDK4/6-RB pathway is critical for the estrogen action on the cell cycle in ER+ breast cancer [8, 9, 17–19].

Optimization of pharmacological CDK4/6 inhibitors has resulted in three orally administered inhibitors: palbociclib [20], abemaciclib [21] and ribociclib [22]. Palbociclib was initially reported to inhibit cell proliferation of ER+/HER2- breast cancer cell lines and in patients who had progressed on endocrine therapy [18, 23]. Progression free survival (PFS) of palbociclib, in combination with endocrine therapy, in ER+/HER2- breast cancer patients has been reported in PALOMA-1 [23], PALOMA-2 [24] and PALOMA-3 [25, 26]. In all demographic subgroups, combination with endocrine therapy had greater PFS than either of the inhibitor alone [23–25]. Thus, clinical trials have established that CDK4/6 inhibitors target luminal breast cancer cells which express ER. However, PALOMA-3 study revealed that hormone-receptor expression level does not affect treatment response in HR+/HER2- women who had progressed on previous endocrine therapy; thus, implying that activated ER required for growth, rather than its level, is requisite for the activity of CDK4/6 inhibitors.

In this preclinical study, model systems were used to demonstrate that cells, expressing a transcriptionally functional ER, which is not linked to cell proliferation or cell cycle progression, do not respond to the treatment of CDK4/6 inhibitors. Herein, we also report the significance of ER, involved in cell cycle progression, and its expression level in the breast cancer response to palbociclib including single cell analysis from patient derived xenograft (PDX) tumor models that are dependent on estrogen for growth. Therefore, our aim was to define the need of ER in cell cycle progression which can identify tumors that will respond effectively to these inhibitors.

## RESULTS

### Cell cycle-driven ER is required for palbociclib inhibition of cell proliferation

**Figure 1:**
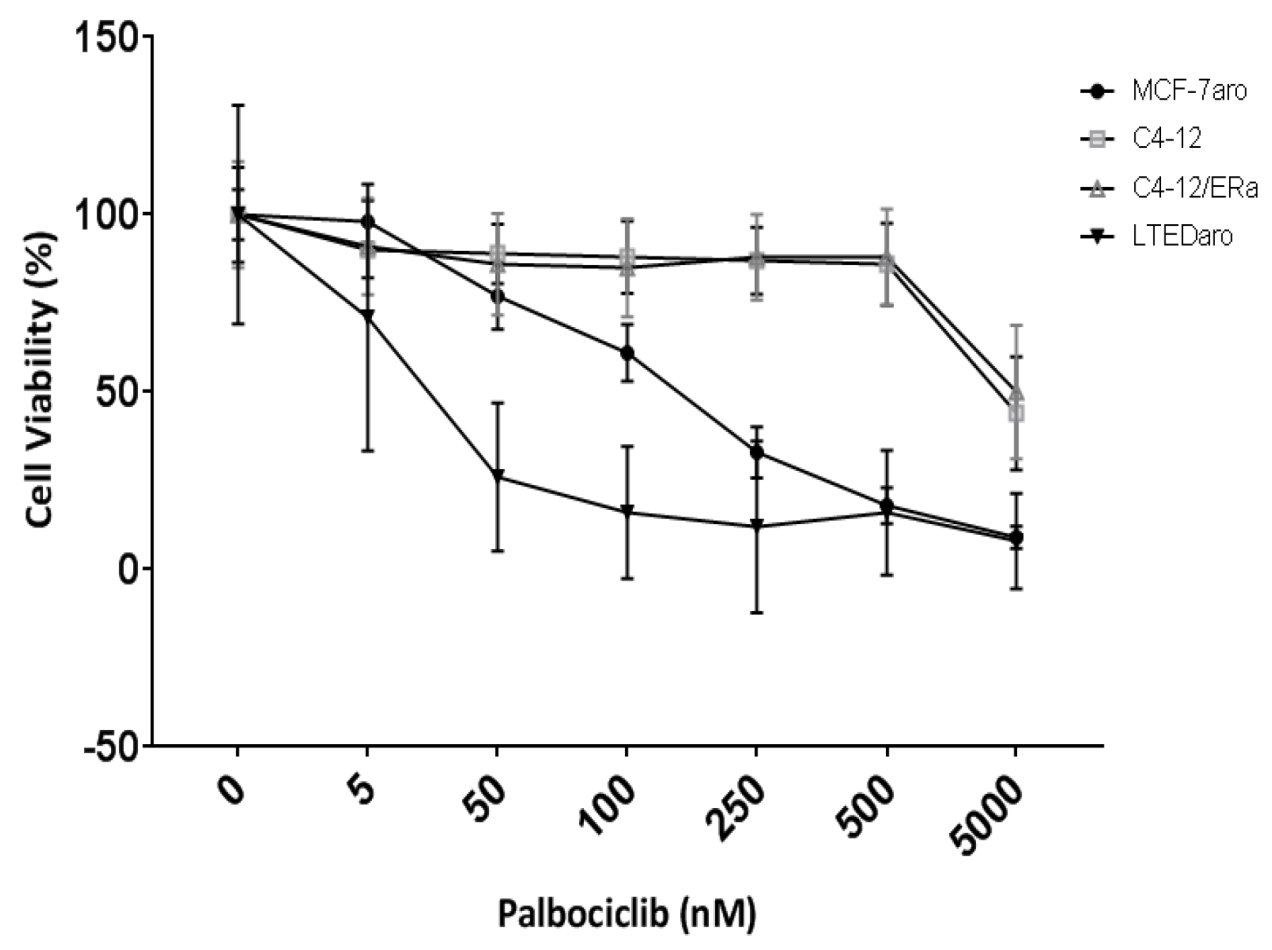
Palbociclib response is dependent on ER which drives cell proliferation Five day cell viability of palbociclib treated cells. Control treatments are testosterone for MCF-7aro cell line and DMSO for the other cell lines; designated as 0nM of inhibitor. Each treatment was performed in three replicates.

In order to elucidate the impact cell cycle-driven ER can have on palbociclib response, we utilized the C4-12 cell line which is a variant of the MCF-7 cell line but lacks an endogenous ERα [27]. We generated a stably transfected ERα (C4-12/ERα) cell line which has a transcriptionally functional ER [28] but does not need estrogen for cell growth (Supplementary Figure 1). CDK4/6 inhibitors did not affect the proliferation of C4-12/ERα (Figure 1), indicating that proliferation of these cells does not require ER even though they have biologically active. As a comparison, we used the MCF-7aro cell line, which requires estrogen for cell proliferation (Figure 1). As expected, palbociclib reduced cell proliferation of MCF-7aro cells and even the long-term estrogen deprived (LTEDaro) cells, which have a constitutively active ER involved in signaling transduction pathways and are involved in cell cycle progression.

Our results indicate that a transcriptionally functional ER involved in cell cycle progression is a requirement for the response to palbociclib in cells which rely on estrogen-activated ER for growth, e.g., as in the MCF-7aro cells, or in cells which have a constitutively active ER, e.g., LTEDaro cells. Moreover, estrogen increased and palbociclib treatment decreased expression of pRB/RB in the MCF-7aro cell line (Supplementary Figure 2: left panel). In the C4-12/ERα cells, estrogen treatment increased protein expression of RB but did not significantly alter that of pRB (Supplementary Figure 2: middle panel; lane 2). Thus, for the response of palbociclib treatment, these experiments show the following: 1) the requirement of a transcriptionally functional ER in the cyclin D1-CDK4/6-RB pathway; 2) the dependency of this activated ER for growth; and 3) the presence of intact RB in these cells. Therefore, ER positivity of the tumor specimen is not sufficient to predict the response of CDK4/6 inhibitors.

### The molecular mechanisms associated with palbociclib treatment in HR+ breast cancer cell lines

To study the role of ER in cell cycle progression and to identify the key molecules that play an important role in this regulation, we compared the gene expression changes between the MCF-7 and C4-12/ERα cell lines upon estrogen stimulation. We observed 29 genes, including 20 estrogen regulated genes (e.g., GREB1, PGR, TIFF1), which were up-regulated due to estrogen (Supplementary Figure 3A and Supplementary Table 2); thus, indicating ER is transcriptionally activated by estrogen in both of these cell lines. Moreover, cell cycle genes (G2/M-phase and checkpoint regulation) were only observed to be up-regulated in the MCF-7 and not in the C4-12/ERα cell line (Supplementary Figure 3B and Supplementary Figure 4A). Thus, the transcriptionally functional ER is not involved in cell cycle progression in the C4-12/ERα cells (Supplementary Figure 3B).

Furthermore, the top canonical pathways most significantly up-regulated with estrogen treatment were involved in the S- and prominently in the G2/M-phase of the cell cycle machinery (Supplementary Figure 4A). Upon estrogen treatment, the G1/S-phase, CDKs and their cyclin partners increase; while p21 inhibitor decreases to allow for cell cycle progression. We observed up-regulation of checkpoint (CHEK1 and CHEK2) and G2/M-phase genes such as the AURKA/BORA complex and PLK1, which induce cyclin B1 aided cell cycle progression (Supplementary Figure 4A). These changes observed in this and other laboratories are indicative of estrogen treatment aiding cell cycle progression with key influences at the G2/M-phase and checkpoint regulation. Also, an *in silico* microarray [29–31] analysis, using the MCF-7 cell line, demonstrated that estrogen modulates all phases of cell cycle machinery, with majority of impact on G2/M-phase and cell cycle checkpoint genes (Supplementary Figure 4B).

**Figure 2:**
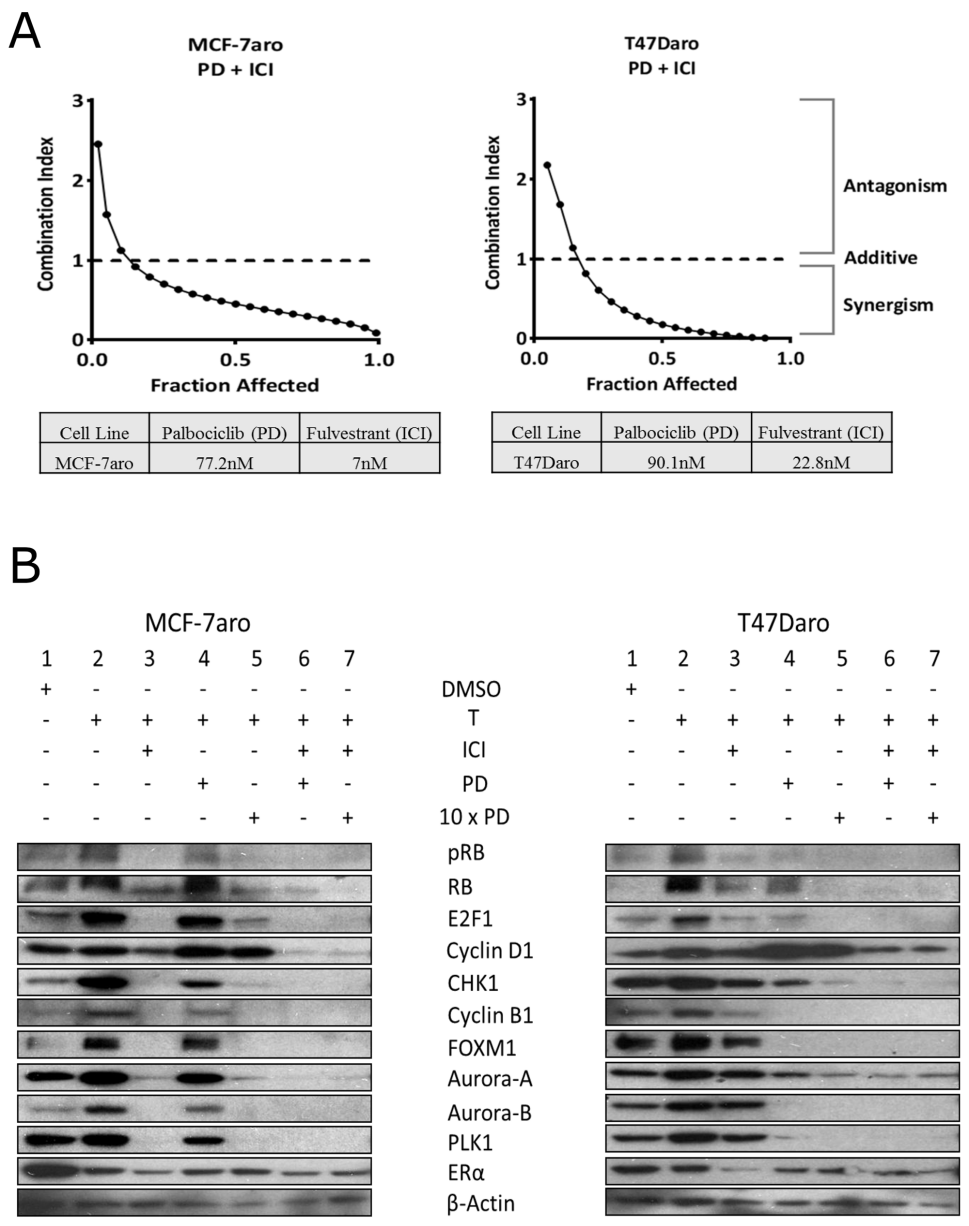
Synergism of palbociclib with ICI in HR+/endocrine therapy responsive cell lines **(A)** Cells were treated with palbociclib (PD) and ICI at ratios based on their IC50 concentrations for 48 hours. Fraction affected was analyzed with CalcuSyn dose effect analysis software. Synergy was observed for concentrations below a combination index (CI) of one. **(B)** Western blot analysis shows palbociclib targets pRB/RB and G2/M-phase proteins after 48 hour treatment. Combination with ICI treatment exhibits significant cell cycle protein reduction versus single treatment. Concentrations of inhibitors used were the IC-50 values.

Clinical data indicates high PFS when palbociclib is used in combination with letrozole or ICI (fulvestrant) in postmenopausal, advanced breast cancer patients [23]. Thus, to determine whether the inhibitory effects on the cell cycle are the key regulatory pathways for combination therapy, we performed the experiment using our HR+ cell line models (MCF-7aro and T47Daro) [32] as proof of concept. Synergism was observed when ICI was combined with palbociclib (Figure 2A). Moreover, we performed cell cycle analysis using the MCF-7aro cells to confirm that testosterone (converted to estrogen) drives cell cycle from G1 to S-phase [8], and palbociclib and ICI inhibit this progression. The percentage of cells in S-phase increased with testosterone treatment (2.2% versus 17.2%). In the presence of ICI, the cells exhibited suppression of the G1/S-phase (94.1% to 0.8%). In addition, combination of palbociclib with ICI indicated a greater cell cycle inhibition at the G1/S-phase transition versus palbociclib alone (93.7% to 0.7% versus 79.7% to 9.5%, respectively) (Supplementary Table 1); thus, providing a mechanistic view on the current treatment regimen of CDK4/6 inhibitors in combination with endocrine therapies.

Through Western blot analysis, we confirmed estrogen (converted from testosterone by the aromatase enzyme) increased the expression of cell cycle proteins while ICI exhibited significant protein reduction in MCF-7aro and to a lesser degree in T47Daro (Figure 2B: lane 2 vs. lane 3). ICI reduced the expression of pRB, E2F1, cyclin D1 and ER protein in both HR+ cell lines (Figure 2B: lane 3). In MCF-7aro, ICI also reduced G2/M-phase protein expression (CHK1, cyclin B1, FOXM1, Aurora-A and B and PLK1) but minimally in T47Daro. On the other hand, palbociclib was found to be more effective in inhibiting protein expression of cell cycle molecules in T47Daro versus MCF-7aro (Figure 2B: lane 4). In MCF-7aro, palbociclib inhibited pRB but had no effect on other cell cycle proteins. When ICI was co-treated with palbociclib, the cell cycle protein expressions reduced synergistically (Figure 2B: lane 4 vs. 6) in both cell lines. Moreover, increase of cyclin D1 protein expression upon treatment was observed prominently in T47Daro, and it has been reported to be due to an active mTOR signaling pathway [33]. Also, reduction in RB levels, post palbociclib treatment, has been documented in other laboratories [34]. MCF-7aro and T47Daro cells responded differently in reducing expression of cell cycle proteins E2F1, cyclin B1, FOXM1, Aurora-A and B and PLK1 post palbociclib treatment, and this could be attributed to the inherent differences between the cell lines. Such results support that the response differences using single drug can be overcome through combined treatment of two drugs.

### G2/M-phase molecular changes associated with treatment of CDK4/6 inhibitors

**Figure 3:**
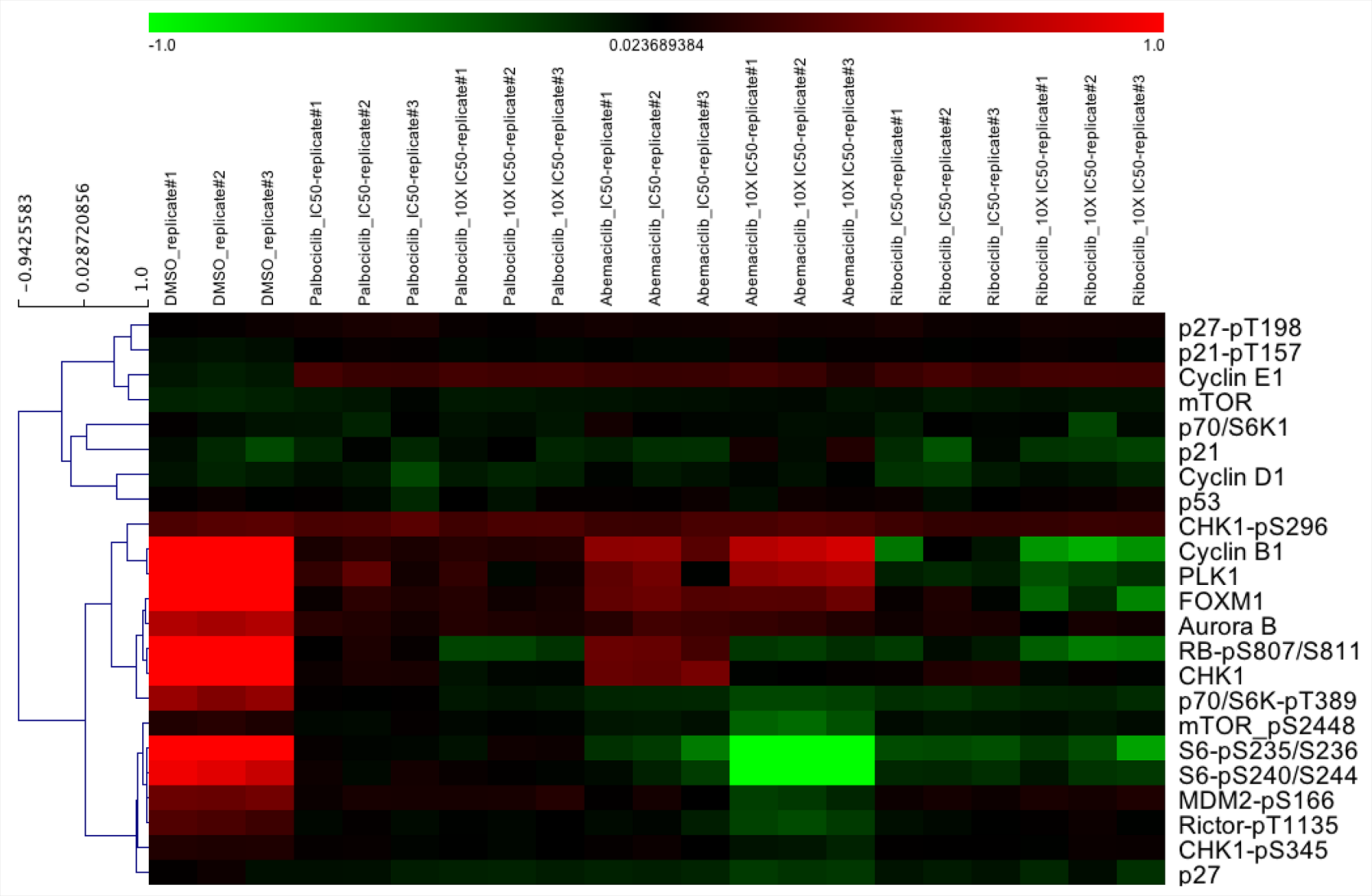
Cell cycle and mTOR signaling pathways are affected by CDK4/6 inhibitors MCF-7aro cells were treated with palbociclib, abemaciclib and ribociclib at their IC50 and 10x-IC50 values for 5 days. Down-regulation of cell cycle proteins and mTOR signaling pathways were observed by RPPA analysis.

In order to analyze the molecular mechanisms of CDK4/6 inhibitor treatment, which have not yet been fully compared among the three inhibitors (palbociclib, abemaciclib, and ribociclib) using an identical model system, we performed a Reverse Phase Protein Microarray (RPPA) utilizing a HR+/aromatase-positive cell line (MCF-7aro). Since all three inhibitors are FDA approved but their clinical response is not identical to each other [35], our goal was to compare the differences of their molecular mechanism of action to each other. Considering the differences in their potencies (IC50 values), abemaciclib (24.2nM) > palbociclib (77.2nM) > ribociclib (234nM), RPPA analysis was performed with IC50 and to maximize protein suppression, 10x-IC50 concentrations (Figure 3).

When all inhibitors were compared in the same set of experiments, they showed down-regulation of RB phosphorylation (i.e., pRB), while abemaciclib showed less inhibition. Expression of cyclin D1, p53, checkpoint kinase 1 (CHK1) and CDK inhibitors (p21 and p27) did not change. Subtle differences were observed among the three inhibitors. G2/M-phase cell cycle proteins such as cyclin B1, PLK1, FOXM1 and Aurora-B were down-regulated more prominently by ribociclib. Moreover, comparing abemaciclib to palbociclib and ribociclib, we observed greater down-regulation of the mTOR pathway (e.g., phospho-mTOR, p70-S6K and S6) than that of the G2/M-phase proteins. Also, CDK4/6 inhibition resulted in the elevation of cyclin E1 levels. Since, cyclin E1/CDK2 also phosphorylates RB; thus, cyclin E1 could serve as an alternative mechanism to aid cell cycle progression leading to CDK4/6 inhibitor resistance [36].

Western blotting experiments were performed on each inhibitor to verify the RPPA results; minor differences are evident, such as abemaciclib treatment (10x-IC50) showed less effect on the G2/M-phase proteins (RPPA) than the other two compounds. The suppression could be observed when abemaciclib was examined individually in the Western blotting results (Figure 3 and Supplementary Figure 5). We utilized palbociclib mainly in this study because it was the first to be FDA approved and the most widely used in the clinic.

### PDX tumor models treated with palbociclib exhibit altered cell cycle protein expression

Because breast cancer tumors are heterogeneous, translational preclinical studies using PDX, as the most clinical relevant tumor model, is critical to answer clinically relevant questions including the mechanism of drug response. Such model offers the possibility to examine the effects of CDK4/6 inhibitors at the single-cell level. COH-SC31, generated in our laboratory, is a unique PDX tumor model. While it is triple positive (ER/PR/HER2), it is also Herceptin resistant; thus, it completely depends on estrogen for growth [37]. Thus, this is a relevant model to demonstrate the molecular action of palbociclib because it depends on activated ER for growth, although it is HER2-positive.

**Figure 4:**
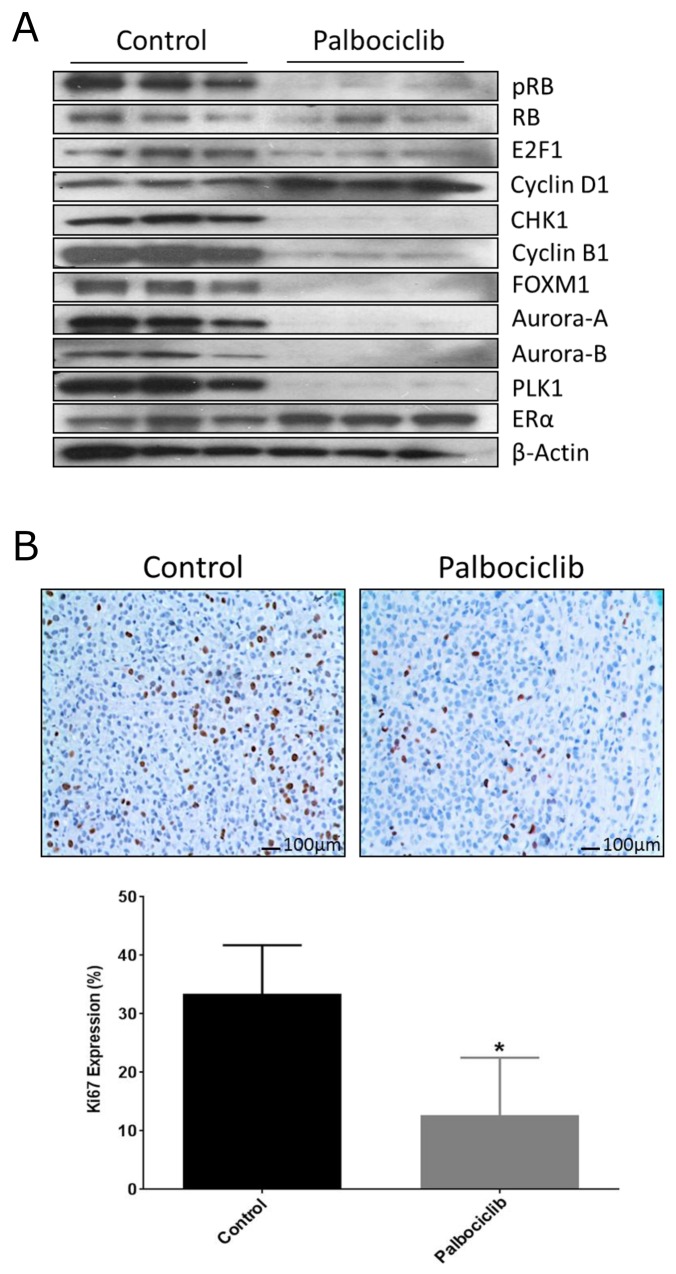

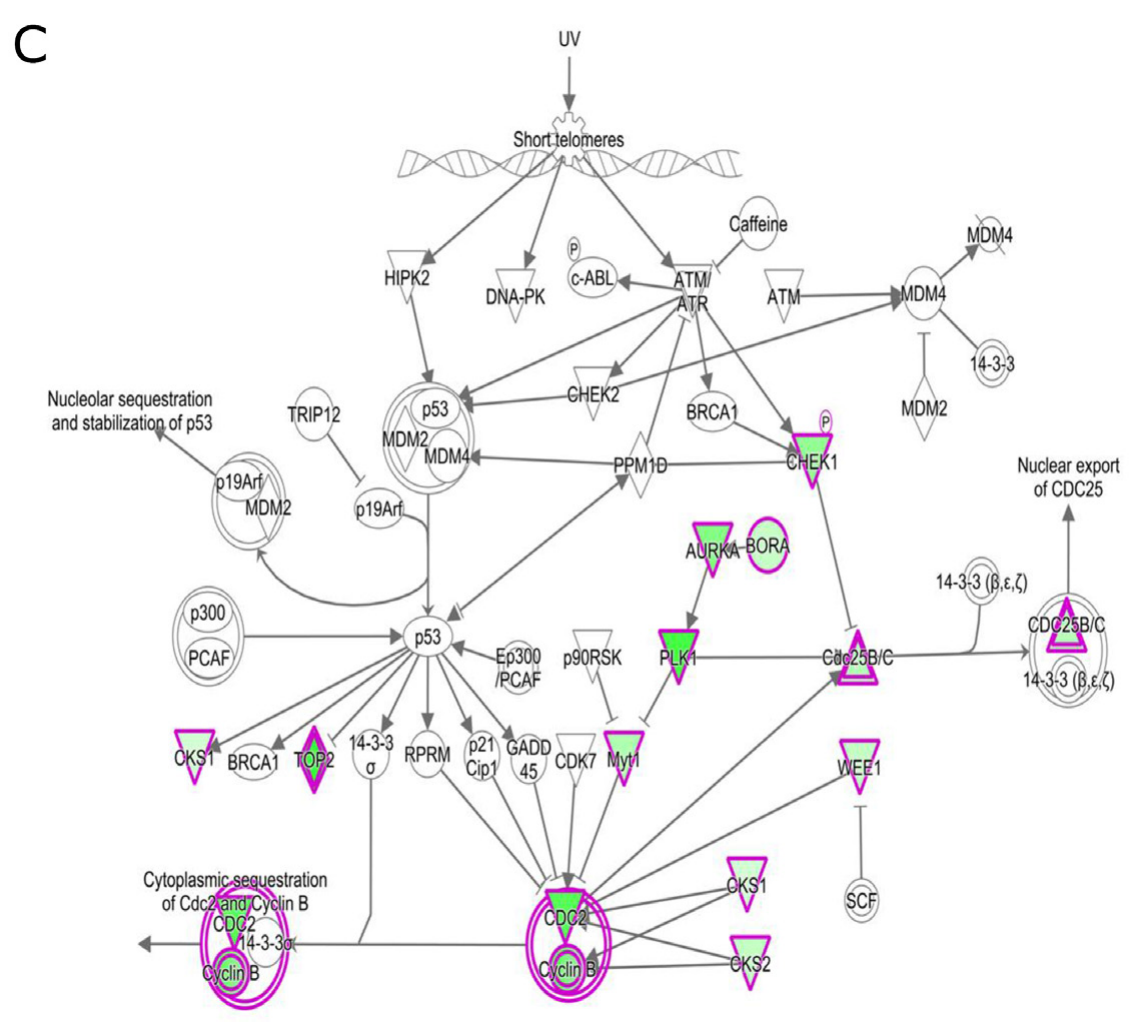
Palbociclib treatment targets cell cycle networks in PDX tumor **(A)** PDX COH-SC31 (ER+/PR+/HER2+) tumor model treated for 3 days with palbociclib shows treatment reduced G1/S- and G2/M-phase proteins. **(B)** Treatment significantly decreased Ki-67 cell proliferation protein expression. ^*^ p< 0.05 **(C)** Gene expression network array shows reduction of G2/M-phase protein pathways with palbociclib treatment: up-regulated (red), down-regulated (green) and unmodified genes (white).

We performed a 3 day palbociclib study and observed reduction of pRB/RB, E2F1, CHK1 and G2/M-phase cell cycle protein expression while cyclin D1 and ER levels were not affected (Figure 4A), behaving identical to the MCF-7aro cells treated with the same drug. Furthermore, IHC analysis of Ki67 expressing cells indicated that palbociclib significantly decreased cell proliferation (Figure 4B). These results were confirmed with another estrogen-responsive breast cancer PDX model (GS4), and we observed similar pattern of cell cycle protein inhibition with palbociclib treatment (Supplementary Figure 6). Furthermore, RNA-seq analyses of COH-SC31 revealed the top canonical pathways most significantly down-regulated by palbociclib were prominently in the G2/M-phase of the cell cycle machinery (CHEK1 and CHEK2, AURKA/BORA complex, PLK1 and CDK1/cyclin B) (Figure 4C). Thus, palbociclib treatment results show similar down-regulation of cell cycle protein expression *in vitro* (Figure 2B) and also in the PDX tumor models (Figure 4A and Supplementary Figure 6).

### Single cell analysis reveals cell cycle genes are altered post palbociclib treatment in cells with high expressing ER

To authentically reflect the innate heterogeneity of a tumor, PDX breast cancer model COH-SC31 was considered a valuable tool to test how the levels of ER, involved in cell cycle progression, affect the tumor response to CDK4/6 inhibitors at the single cell level. In order to isolate intact epithelial cells, we took advantage that this tumor is triple positive (ER/PR/HER2), growth dependent on estrogen and it is Herceptin resistant with homogenous HER2 expression [37].

**Figure 5:**
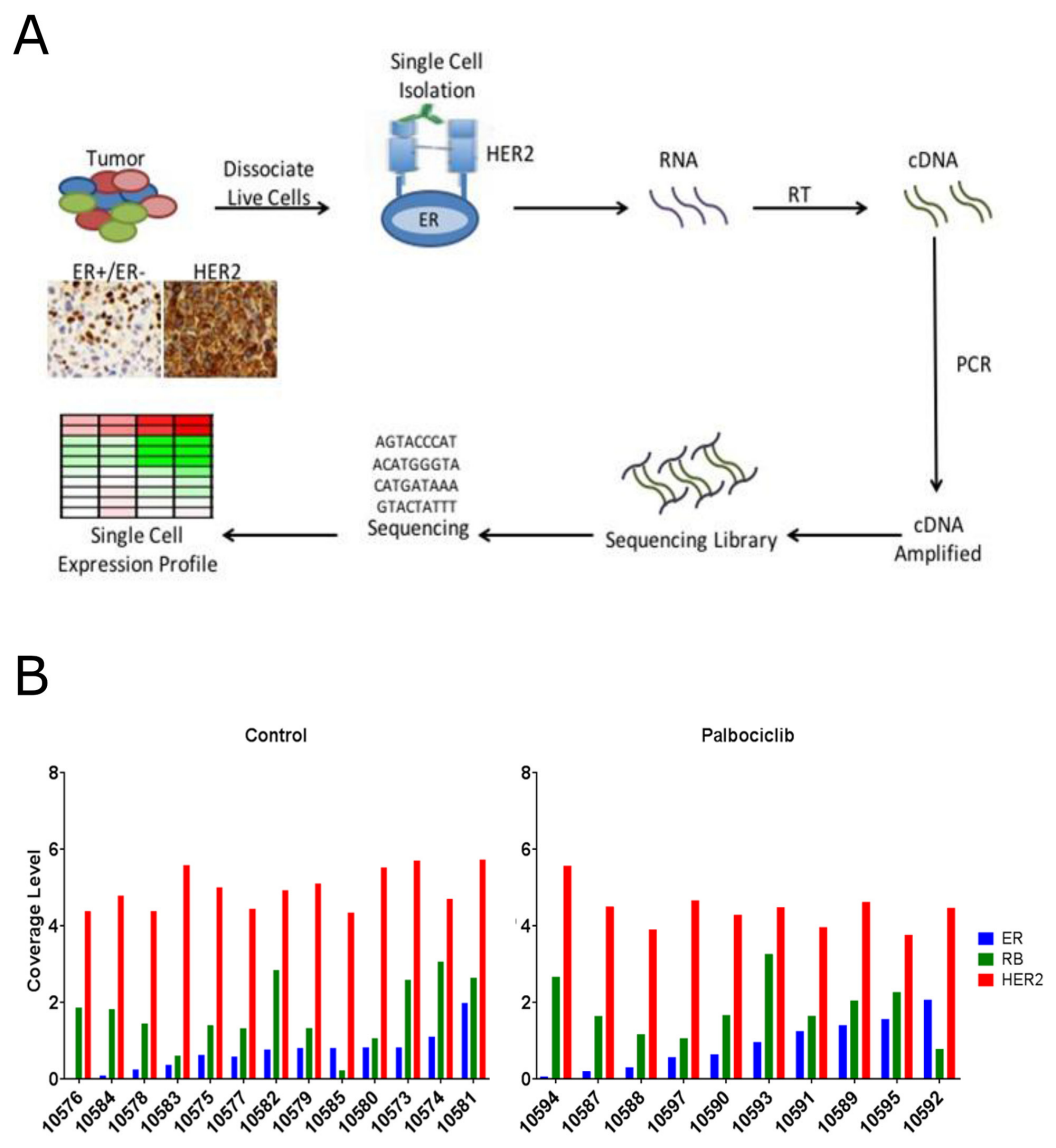
Palbociclib treatment alters gene expression pathways of single cells isolated from a PDX tumor model **(A)** PDX COH-SC31 (ER+/PR+/HER2+) tumor expresses different levels of ER but similar levels of HER2. Tumor is dissociated into intact live cells and HER2 antibody is used to isolate the single tumor cells with the DEPArray technique. **(B)** Sequencing coverage levels in control and treated single cells indicate high and low ER and RB groups of cells based on the homogeneous expression of HER2 in each individual cell. Cut-off sequence coverage read levels were set for 0.8 (ER) and 1.5 (RB).

Since ER is intracellular and selecting for it would jeopardize the intactness of the isolated RNA and the COH-SC31 tumor displays uniform expression of HER2, it was feasible and unique to utilize the HER2 receptor and select individual tumor cell. Using the DEPArray technique, tumors were excised, digested and isolated into intact single cells and labeled with an antibody for the extracellular portion of the HER2 receptor (Figure 5A). Out of 30 isolated live HER2+ epithelial cells, 13 and 10 individual cells from the control and palbociclib-treated tumors, respectively, passed quality control and were used for single cell RNA-seq analysis. Single cell preparations had similar levels of HER2 expression; thus, the DEPArray approach successfully isolated HER2+ cells (Figure 5B). Also, single cell preparations exhibited variation of ER and RB expression which is expected due to the heterogeneity in the tumor (Figure 5B). This observation also agreed with the IHC staining of this PDX which is approximately 40-50% ER+ [37]. Moreover, as ER coverage reads increased, there is no correlation with the RB expression; thus, ER and RB are two independent factors in the cell cycle (Figure 5B).

To analyze gene expression profiles within individual cells and to address how ER and RB affect the action of palbociclib, the single cell preparations of the control and palbociclib groups were separated into two expression subgroups: ER^high/low^ and RB^high/low^. The high and low groups were determined during the sequencing step by setting a limit with the coverage reads to ensure each group contained similar mean of ER or RB expression (Figure 5B). Analysis of ER^high^ versus ER^low^ in the control group revealed top 5 up-regulated networks in ER^high^ were all associated with cell cycle regulation, confirming that estrogen, through the interaction with ER, drives the cell cycle in these ER+ cells/tumors (Table 1: top left panel). In another set of analysis, ER^high^ in the control versus that in the treatment group, palbociclib resulted in the down-regulation of cell cycle genes (Table 1: middle left panel). Moreover, ER^low^ in the control versus that in the treatment group, palbociclib treatment did not affect cell cycle genes (Table 1: lower left panel).

**Table 1:** Gene expression analysis at the single cell level

ER^high^: up-regulated pathways in untreated cells		RB^high^: up-regulated pathways in untreated cells	
Pathways	p-value	Pathways	p-value
M phase	1.64E-48	M phase	1.44E-18
cell cycle phase	2.05E-45	cell division	1.46E-17
nuclear division	8.14E-44	cell cycle phase	4.77E-16
mitosis	8.14E-44	mitosis	6.62E-16
M phase of mitotic cell cycle	2.23E-43	nuclear division	6.62E-16

ER^high^: down-regulated pathways in treated cells		RB^high^: down-regulated pathways in treated cells	
Pathways	p-value	Pathways	p-value
M phase	4.97E-17	M phase	3.36E-10
cell cycle	2.04E-16	cell cycle	1.20E-09
cell cycle phase	2.01E-14	cell cycle phase	5.79E-09
cell cycle process	3.13E-14	nuclear division	1.76E-08
mitotic cell cycle	7.86E-14	mitosis	1.76E-08

ER^low^: down-regulated pathways in treated cells		RB^low^: down-regulated pathways in treated cells	
Pathways	p-value	Pathways	p-value
regulation of protein complex disassembly	3.11E-04	peptidyl-lysine modification	3.99E-03
regulation of cytoskeleton organization	1.08E-03	maintenance of protein location in cell	4.97E-03
regulation of protein polymerization	1.18E-03	memory	6.47E-03
negative regulation of protein complex disassembly	1.30E-03	maintenance of protein location	6.70E-03
barbed-end actin filament capping	1.32E-03	protein acylation	6.97E-03

Furthermore, analysis of RB^high^ versus RB^low^ revealed top 5 up-regulated networks in RB^high^ were all associated with cell cycle regulation, confirming that RB also drives the cell cycle in ER+/RB+ cells/tumors (Table 1: top right panel). In RB^high^, unlike RB^low^, palbociclib treatment resulted in the down-regulation of cell cycle genes (Table 1: middle right panel and lower right panel). Notably, the p-values were derived from a hypergeometric distribution; thus, the p-value depends on number of differentially expressed genes in the pathway and not on the number of samples in each group. These 2-way comparison results of ER and RB (high and low levels) indicate that both are important for cell cycle progression which is inhibited by palbociclib. However, ER plays a significant role as indicated by the smaller p-values (1.64E-48) versus RB (1.44E-18) (Table 1: top two panels). These gene expression analysis of single cell preparations confirmed that ER drives cell cycle progression in ER^high^ cells; CDK4/6 inhibitors, such as palbociclib, are effective mainly in ER^high^ cells which target the cell cycle; and these inhibitors target the ER+/HER2+ cancer when the tumors express high levels of ER and use the ER pathway for cell growth.

## DISCUSSION

Significant proportion of breast cancers exhibit dysregulation of cell cycle networks, specifically the cyclin D1-CDK4/6-RB pathway [38], and antiestrogens have been shown to inhibit the phosphorylation of RB causing G1 arrest [18, 20, 39]. ER+ breast cancer depends on the cyclin D1-CDK4/6-RB pathway for growth and this pathway is targeted by CDK4/6 inhibitors [18, 40]. Combining CDK4/6 inhibitors with endocrine therapies have exhibited an increase of PFS in ER+/HER2- metastatic breast cancer patients [25].

In this manuscript, we showed the C4-12 cell line, which is a variant of the MCF-7 cell line and lacks endogenous ER did not respond to estrogen (Supplementary Figure 1) or palbociclib treatment (Figure 1). We have established that C4-12/ERα cells do not require estrogen for cell growth (Supplementary Figure 1) but ER is activated by estrogen as seen with the up-regulation of ER regulated genes such as GREB1, TFF1 and PGR, which is also observed in the estrogen dependent MCF-7 cell line (Supplementary Table 2). Analysis of C4-12/ERα cells shows no change in the G2/M-phase or checkpoint regulation genes (Supplementary Figure 3B); thus, the transcriptionally functional ER in this cell line [28] is not involved in cell cycle progression. Moreover, even though this cell line expresses RB, which alone is not a predictive factor of palbociclib response [18, 20, 40–42], a transcriptionally functional ER involved in cell cycle progression is required for the inhibitor response. Furthermore, LTEDaro cells are hormone-independent with a constitutively active ER which does not depend on estrogen for activation [43]; exhibit ligand-independent recruitment of ER to ER-responsive gene promoters [43]; have developed cross-talk with alternative growth factor pathways [5, 6, 44]; and it has been concluded that ER-mediated signaling pathways are responsible for cell growth [5, 43, 44]. Thus, supporting the findings from PALOMA-3 [25, 26], LTEDaro serves as a model of endocrine-therapy resistant cell line which responds to palbociclib treatment due to its constitutively active ER signaling pathways (Figure 1).

Monotherapy treatment of CDK4/6 inhibitors had minimal inhibition on G2/M-phase proteins (Figure 3) but when used in combination with endocrine therapy (ICI), synergism was more effective on the inhibition of these proteins (Figure 2B and Supplementary Figure 5). We and others in the field attribute this inhibition to the RB/E2F1 loss-of-function post treatment [20, 41] which targets multitude of G1/S/G2-phase proteins critical for cell cycle progression [45]. Moreover, we showed synergism as the mechanism between the estrogen antagonist ICI and CDK4/6 inhibitors (Figure 2 and Supplementary Figure 5). Also, as validated by our laboratory and others, in HR+/endocrine therapy responsive cell line models, estrogen increased cell cycle network RNA and protein expression (Figure 2B: lane 2 and Supplementary Figure 4A) [8]. Thus, this synergistic effect, on the cell cycle protein expressions, is through the ER [40]. Moreover, there were subtle differences among the three CDK4/6 inhibitors (Figure 3). As compared to the control, G2/M-phase proteins were down-regulated mostly by ribociclib and palbociclib, and the mTOR signaling pathways were altered mainly by abemaciclib. As observed by Goel *et al.*, abemaciclib treatment attenuates mTOR function and this was clearly evident in the RPPA results [35, 42, 46] (Figure 3).

Most mechanistic studies have been carried out in breast cancer cell lines, which are clonally selected, and since breast tumors are heterogeneous, we utilized two PDX tumor models to elucidate the role of transcriptionally functional ER in the CDK4/6 inhibitor response. In established ER+ PDX tumor models, like clinical tumors, we and others have noticed that ER expression levels do not always correlate with the estrogen dependency for tumor growth [47]. This characteristic is attributed to inter- and intra-tumoral heterogeneity leading to different patterns of estrogen response on tumor growth [47–49]. Furthermore, the PDX model, which authentically represents human breast cancer, is the most feasible to study the ER action in each single cell. Unlike cell line xenografts, PDX models recapitulate the tumor microenvironment and the differing ER expression levels; thus, single cell isolation from a heterogeneous tumor will aid in elucidating the inhibitor’s mechanism of action on heterogeneous ER expressing tumor cells [50, 51].

The study was tailored for 3 day palbociclib treatment because the direct effect of an inhibitor, on its molecular targets, can best be detected during the half-life of the drug [20, 52, 53]. Similar to the *in vitro* results, the *in vivo* results showed inhibition of checkpoint regulators, G1/S- and G2/M-phase cell cycle protein and RNA (Figure 4A, 4C and Supplementary Figure 6), and significant reduction of Ki67 protein expression (Figure 4B). Thus, palbociclib targets proliferation of ER+ tumors through inhibition of ER-mediated cell cycle progression. Any defects in the G1/S or G2/M-phase proteins could result in the resistance of CDK4/6 inhibitor treatments.

Individual cells in clinically defined ER+ tumors have different levels of ER expression. COH-SC31 tumor exhibits 40-50% ER+ cells; thus, 50-60% of the cells are defined as ER negative using IHC analysis [37]. IHC detected ER expression levels are not a predictive marker for CDK4/6 inhibitor response, as suggested by the different response to estrogen in ER+ PDX tumor models [49]. We hypothesized that the inhibition of ER dependent cell growth pathways are crucial for palbociclib treatment; therefore, the individual cells with different ER expressions are valuable tools to test our hypothesis. To uncover the first mechanistic evidence, we utilized DEPArray technology and isolated functionally active cancer cells through cell surface HER2 labeling. We determined the molecular profile of each live single cell population as being ER+ (high) or ER- (low) and performed bioinformatics analysis on these single cell preparations to interpret the function of altered genes and their biological pathways.

Variation of ER and RB expression was observed in each single cell but the levels of expression did not correlate to each other (Figure 5B). High expression of ER in the single cells revealed that the top up-regulated networks were associated with cell cycle regulation, and treatment with palbociclib specifically targeted and suppressed these cell cycle networks. In stark contrast, the ER^low^ single cells, in the treated group, did not show suppression of cell cycle networks (Table 1). Moreover, in a separate analysis of these single cell preparations, variation of RB expression levels (RB^high^ versus RB^low^), which were independent of the ER expression levels, displayed similar gene ontology networks as the ER (Table 1). Therefore, ER and RB are two independent markers that drive cell cycle progression in functional ER+/RB+ cells/tumors. Through the single cell preparations, the effect of palbociclib on cell cycle networks, in high expressing ER and RB single cells, can be clearly observed. But this is not observed in the low expressing ER and RB cells.

These single cell preparations, along with their differently expressed gene ontology pathways, undoubtedly indicate that both ER and RB are important to the cell cycle progression; however, ER has a significant role as indicated by the smaller p-values (1.64E-48) versus RB (1.44E-18). Thus, cell cycle-driven/functional ER is critical for the success of CDK4/6 inhibitor treatments. These exciting results clearly demonstrate that CDK4/6 inhibitors, such as palbociclib, are effective mainly in ER expressing cells which rely on the ER-cyclin D1-CDK4/6-RB pathway for growth.

In conclusion, this preclinical study has demonstrated the importance of the active ER-cyclin D1-CDK4/6-RB pathway for an effective response of CDK4/6 inhibitors. Through experiments using our CDK4/6 inhibitor-responsive and -resistant cell lines, as well as PDX models, attempts have been made to search for predictive markers for such treatment. While cell cycle genes/proteins downstream of ER-cyclin D1-CDK4/6-RB pathway would be logical candidates, we have not been able to identify markers with convincing experimental evidence. ER and RB are still the best markers, but their levels and activities would matter in the response to CDK4/6 inhibitor treatment.

## MATERIALS AND METHODS

### Cell lines

MCF-7 and MCF-7aro [32] were cultured in phenol red MEM1x; LTEDaro [6] was cultured in phenol red-free MEM1x; and T47Daro [32] was cultured in phenol red RPMI-1640. Cell culture media was supplemented with 10% FBS or 10% charcoal/dextran treated FBS for LTEDaro, 2 mM L-glutamine, and 50mg/ml of G418 for the MCF-7aro, LTEDaro and T47Daro cell lines. C4-12 was cultured in phenol red-free high glucose DMEM with 10% charcoal/dextran treated FBS [27]. C4-12/ERα cell line was generated as previously reported [28]. Culture media were supplemented with 1 mM sodium pyruvate and 100 U/mL penicillin-streptomycin. Cell lines were authenticated at the Integrative Genomics Core of the City of Hope (City of Hope, Duarte, CA).

### Reagents

Testosterone and 17β-estradiol (estrogen) were purchased from Sigma (Sigma Chemical, St. Louis, MO) and fulvestrant (ICI 182780) was purchased from Tocris (Ellisville, MO). Palbociclib (PD0332991), abemaciclib (LY2835219), and ribociclib (LEE011) were purchased from Selleckchem (Houston, TX).

### Cell cycle analysis

MCF-7aro cells were hormone starved for 72 hours, in white MEM1X with 10% charcoal-dextran treated FBS, prior to treatment for 48 hours with DMSO, 1nM testosterone, 100nM ICI or the IC50 value of palbociclib (77.2nM). Cells were harvested, stained with Annexin V/propidium iodine and analyzed as previously described [54].

### Determination of IC50 and synergistic studies

IC50 and combination index were calculated by Calcusyn 2.1 software (Biosoft, Cambridge, UK). To determine synergism, cells were treated by palbociclib and ICI either as single agents or in combination at 1/4 IC_50_, 1/2 IC_50_, IC_50_, 2x-IC_50_, and 4x-IC_50_, according to the Chou-Talalay method [55]. Cell viability was assayed with MTT (Sigma, St. Louis) after 5 days when cell growth reached the exponential phase.

### Immunohistochemistry (IHC)

IHC was performed by the Molecular Pathology Core at the City of Hope. Slides were incubated with Ki67 (Dako M7240; Santa Clara, CA) antibody and Ki67 labeling index was performed according to previously published guidelines [56]. Five randomly selected fields were collected at 200× magnification for each sample and images were captured with Olympus DP72 camera (Olympus, Shinjuku, Japan). Ki67 labeling index was presented as the average of three biological replicates using the ImageJ public domain software (developed by Wayne Rasband of the NIH).

### Reverse phase protein microarray (RPPA) analysis

Cells were treated with IC50 or 10x-IC50 concentrations of palbociclib, abemaciclib, and ribociclib for 5 days. The cells were then lysed per the cell line lysate prep (6-well plate) protocol of the University of Texas, MD Anderson Cancer Center RPPA Core Facility-Functional Proteomics (Houston, Texas). Protein expression in these samples was then estimated through RPPA.

### Western blot analysis

Cell lines: 48 hours post inhibitor treatment, protein was extracted with RIPA buffer supplemented with 1mM phenylmethanesulfonyl fluoride (PMSF). PDX tumors: proteins were homogenized with Precellys tissue homogenizer (Bertin Technologies, Wilmington, DE) with RIPA buffer and 1mM PMSF (6500rpm-1run-30sec-30sec pause; Precellys ceramic beads). Protein concentration was determined using the Bradford Protein Assay (BioRad, Hercules, CA). The following antibodies were used: cyclin D1 (DCS-6), E2F1 (KH95), ERα (HC-20), FOXM1 (C-20) and β-actin (I-19R) purchased from Santa Cruz Biotechnology (Dallas, Texas); Aurora-B, CHK1 (2G1D5), cyclin B1 (V152), PLK1 (208G4), RB (4H1) and phosphorylated-RB (S807/811) purchased from Cell Signaling Technology (Beverly, MA); and Aurora-A purchased from EMD Millipore (Temecula, CA). Signal intensity was visualized by ChemiDoc MP Imaging System (Bio-Rad, Hercules, CA).

### Palbociclib studies using patient-derived xenograft (PDX) tumor model

Patient derived xenograft (PDX) tumor models were generated from donated breast cancer tissues from patients at the City of Hope Medical Center. The use of the tissues was approved by the Institutional Review Board (IRB) and animal studies were approved by the Institutional Animal Care and Use Committee (IACUC). A triple positive (COH-SC31:ER+/PR+/HER2+) and a double positive (GS4: ER+/HER2+) breast cancer tumors were implanted into 6-8 week old female NOD/SCID/interleukin-2 receptor gamma chain null (NSG) mice as previously described [48]. Three-mice per treatment group was used for a 3 day biomarker study. The mice were gavaged with palbociclib (150mg/kg/d) or vehicle (PBS). Tumor volume was calculated by π/6 x L x W^2^, and body weight was measured daily prior to treatment.

### Isolation of intact single cells by DEPArray

Tumors were excised and subjected to digestion with Liberase-TH and -TM, washed with staining buffer, stained with HER2 antibody (ab31891; Abcam, Cambridge, MA) and delivered to the City of Hope Integrative Genomics core (City of Hope, Duarte, CA) for further processing. The stained cells were then gently mixed and washed with 1ml manipulation buffer (high glucose DMEM + 10% FBS + 1mM sodium pyruvate + 0.5nM estrogen + 100U/ml penicillin/streptomycin). One ml of manipulation buffer with RNase inhibitor (1 unit/μl) was degassed. The DEPArray A300K cartridge was injected with 830μl manipulation buffer and 13μl sample and loaded into the DEPArray equipment per manufacturer’s instructions. Cell selection was performed by Cell Sorting Execution Start Up, followed by Chip Scan Configuration, Chip Scan and Image Analysis. Single live cells with high HER2/FITC fluorescence and intact DAPI nuclear staining were selected. The selected cells (thirty single cells) were automatically recovered into 0.2ml PCR tubes.

### RNA-sequencing

For tumor RNA-seq analysis: Total RNA from 3 day treatment of COH-SC31 palbociclib treated tumors, and MCF-7 (SRP035276) and C4-12/ERα cells treated for 48 hours with control (DMSO) or 1nM estrogen were extracted using RNeasy Extraction Kit (Qiagen, Alameda, CA) and subjected to RNA-sequencing by the Integrative Genomics Core (City of Hope, Duarte, CA) using an Illumina HiSeq 2500 system following manufacturer’s protocols (Illumina Inc. San Diego, CA).

For MCF-7 and C4-12/ERα cell line RNA-seq analysis: Reads were aligned against the human genome (hg19) using TopHat2 [57]. Read counts were tabulated using htseq-count [58], with UCSC known gene annotations (TxDb.Hsapiens.UCSC. hg19.knownGene [59]). Because the previous dataset had an unstranded library (SRP035276) and the newer dataset had an unstranded library (GSE114260), htseq-count quantified unstranded counts for both datasets. Fold-change values were calculated from Fragments Per Kilobase per Million reads (FPKM) [60], normalized expression values, which were also used for visualization (following a log_2_ transformation). Aligned reads were counted using GenomicRanges [61]. P-values were calculating from raw counts using edgeR [62], and false discovery rate (FDR) values were calculated using the method of Benjamini and Hochberg [63]. Prior to p-value calculation, genes were filtered to only include transcripts with an FPKM expression level of 0.1 (after a rounded log2-transformation) in at least 50% of samples [64] as well as genes that are greater than 150 bp. Genes were defined as differentially expressed if they had a |fold-change| > 1.5 and FDR < 0.05. Additional systems-level analysis was performed in IPA (Ingenuity^®^ Systems, www.ingenuity.com); for the set of genes with the same direction of change for MCF7 and C4-12 cells, the background set of gene was all genes in the IPA database.

For single cell RNA-seq analysis: The single cell collection buffer volume was first reduced to 1μl, the SMART-Seq V4 Ultra Low Input RNA Kit for Sequencing (Clontech, Mountain View, CA) was used for RNA extraction, reverse transcription and double stranded cDNA amplification per manufacturer’s instructions. After cDNA amplification, quality control tests were conducted by qPCR and Bioanalyzer using DNA high Sensitivity Chip (Agilent Technologies, Santa Clara, CA).

### Single cell RNA-seq library preparation, sequencing and data analysis

The cDNA was fragmented by Covaris S220 (Covaris Inc., Woburn, Massachusetts) with the 200bp peak setting. The fragmented cDNA underwent end repair, 3′ ends adenylation and the barcoded adapters (Illumina Inc., San Diego, CA) ligation with Kapa LT library preparation kit (Kapa Biosystems, Wilmington, MA). Ligation products were purified and amplified with a 10-cycle PCR. The prepared libraries were validated using a 2100 Bioanalyzer DNA High Sensitivity chip, and quantified by Qubit Fluorometric Quantitation (Waltham, MA). The library templates were prepared for the sequencing using cBot cluster generation system with HiSeq SR Cluster Kit V4 (Illumina). The sequencing run was performed in a single read mode of 51 cycles of read 1 and 7 cycles of index read using HiSeq 2500 platform with HiSeq SBS Kit V4 (Illumina). Image analysis and base calling were carried out by HiSeq Control Software (HCS) 2.2.38 and Real Time Analysis (RTA) 1.18.61 on the Illumina HiSeq 2500 machine.

Raw sequence reads were mapped to the human genome (hg19) using TopHat (Kim 2013), and the frequency of Refseq genes was counted using customized R scripts. The raw counts were then normalized using the trimmed mean of M values (TMM) and compared using Bioconductor package “edgeR” [62]. Coverage levels were normalized per base coverage, calculated by (normalized counts ^*^ read length 51bp)/gene length, indicating how many reads mapped to the gene adjusted by read length and gene length to the per base resolution. Coverage levels were set as 0.8 and 1.5 for ER and RB, respectively. Coverage levels above and below these specific values we defined as high and low expressing, respectively. Differentially expressed genes were identified if fold change was ≥2, and *P* ≤ 0.05. These differential genes were then imported into Ingenuity Pathway Analysis (IPA^®^, QIAGEN Redwood City, www.qiagen.com/ingenuity) for correspondent pathway and network analyses. GO category enrichment analysis was performed with Database for Annotation, Visualization and Integrated Discovery (DAVID) [65]. Kyoto Encyclopedia of Genes and Genomes (KEGG) and Gene Ontology (GO) corresponding to biological processes (BP-FAT) were used to access the functional analysis of the RNA-seq expression data of the up- and down-regulated genes in the control and treated groups. The fold enrichments, in these analyses, were based off of the abundance of sequencing reads of the control treatment subtracted from the palbociclib treatment, and the count number was for the number of reads mapped to the transcript.

### Statistical analysis

Unpaired (Student’s) *t*-test was performed in Graphpad Prism (La Jolla, CA, USA) to directly compare treatment effects between treatment and vehicle groups. ^*^p<0.05.

## SUPPLEMENTARY MATERIALS FIGURES AND TABLES





## Abbreviations

PDX: patient-derived xenografts
ER: estrogen receptor
PR: progesterone receptor
HER2: human epidermal growth factor receptor 2
RB: retinoblastoma tumor suppressor gene
PFS: progression free survival
