# Supplementary material for: ERα-mediated cell cycle progression is an important requisite for CDK4/6 inhibitor response in HR+ breast cancer
Source: Oncotarget. 2018 Jun 12;9(45):27736–51. doi: 10.18632/oncotarget.25552 (PMC6021239; doi:10.18632/oncotarget.25552)
Supplement: Supplementary file 1 [file oncotarget-09-27736-s001.pdf]

ERα-mediated cell cycle progression is an important requisite for CDK4/6 inhibitor response in HR+ breast cancer

SUPPLEMENTARY MATERIALS

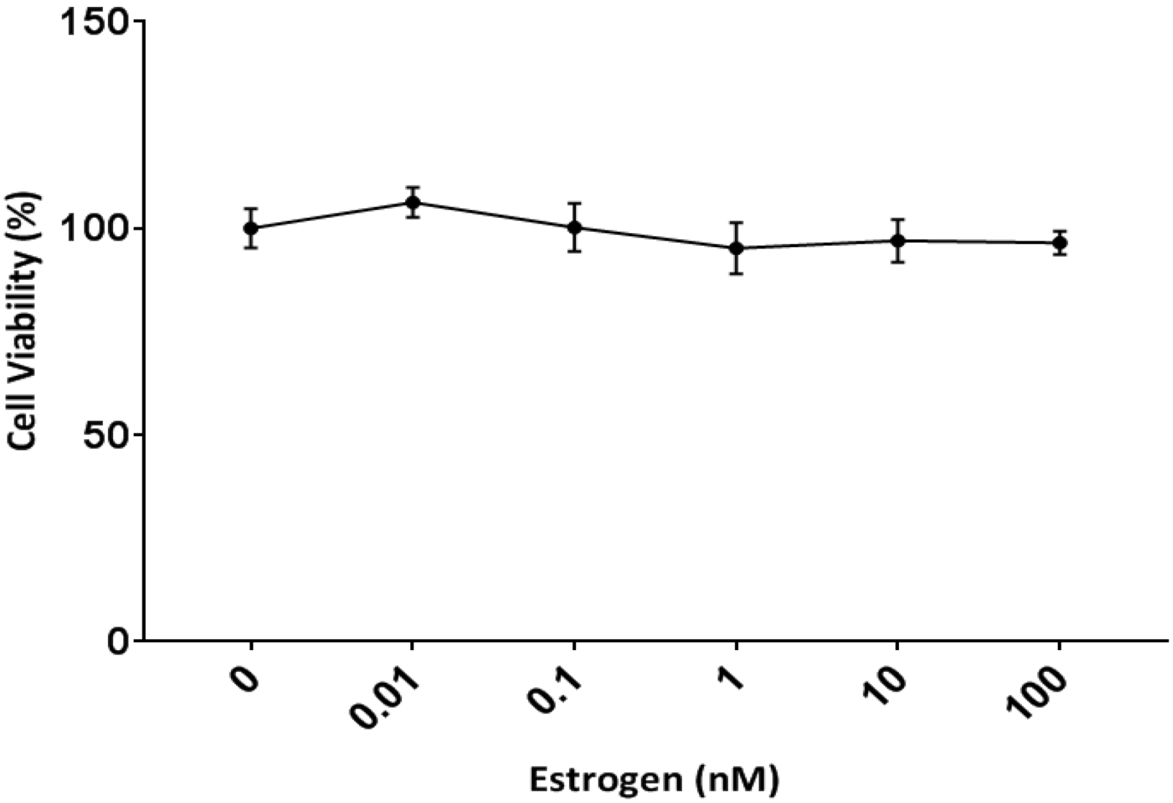

Supplementary Figure 1: Estrogen treatment has no effect on C4-12/ERα cell proliferation. Five day cell viability assessment of estrogen treated cells. Controls treatments are DMSO; designated as 0nM of estrogen. Each treatment was performed in three replicates.

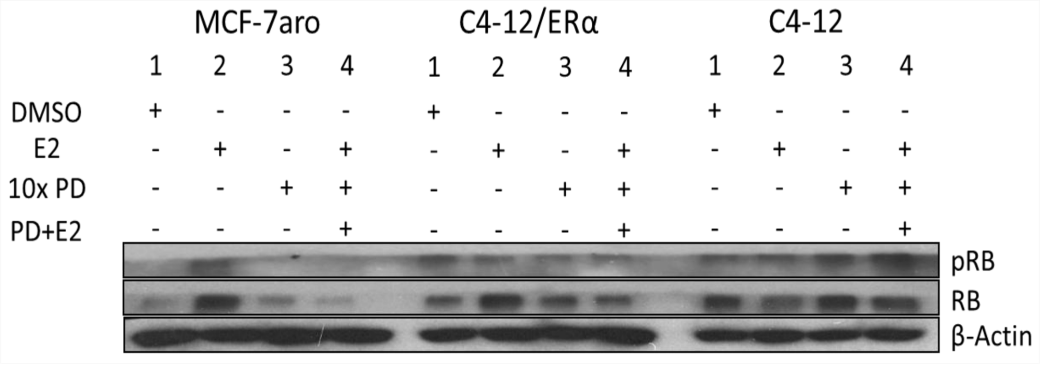

Supplementary Figure 2: Palbociclib treatment targets cells that rely on estrogen for cell cycle progression. Western blot analysis shows protein reduction of pRB/RB in MCF-7aro cells treated 48 hours with palbociclib but no protein reduction was observed in C4-12 or C4-12/ERa cell lines. C4-12/ERa showed minimal increase of RB with estrogen treatment but these cells did not respond to palbociclib treatment. 10x-IC50 concentration of palbociclib was used to maximize the effect on the pRB/RB protein expression.

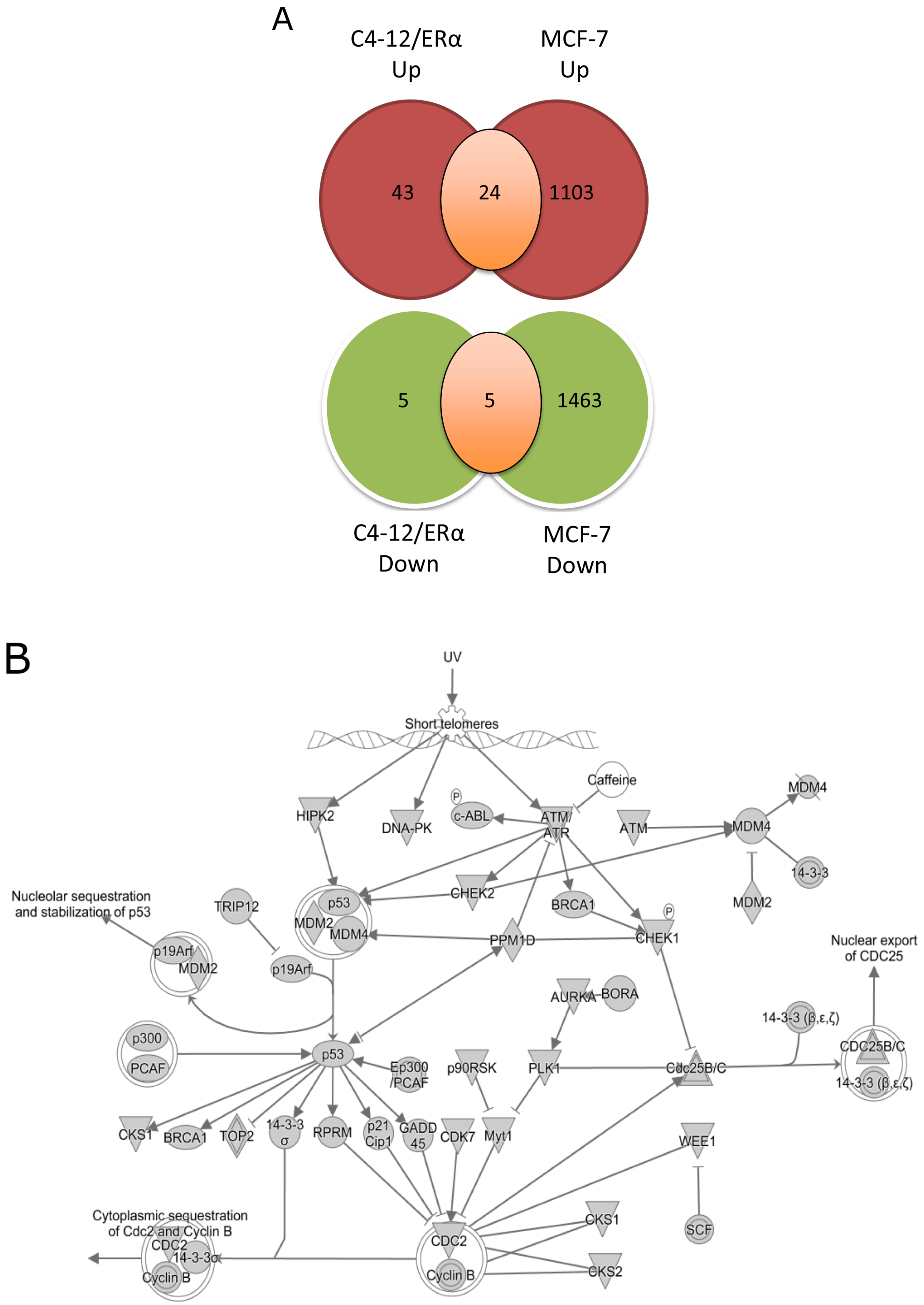

Supplementary Figure 3: Estrogen up-regulates common genes for MCF-7 and C4-12/ERα cells, but it does not up-regulate cell cycle genes in the C4-12/ERα cells. (A) Venn diagrams of MCF-7 and C4-12/ERα cells treated for 48 hours with DMSO or estrogen shows overlapped ER-regulated genes (24 up- and 5 down-regulated); MCF-7 (1103 genes up- and 1463 down-regulated); and C4-12/ERα (43 genes up- and 5 down-regulated). **(B)** Gene expression network array of C4-12/ERα cells treated for 48 hours with DMSO or estrogen shows no change of G2/M-phase genes with estrogen treatment: up-regulated (red/pink), down-regulated (green) and unchanged genes (grey).

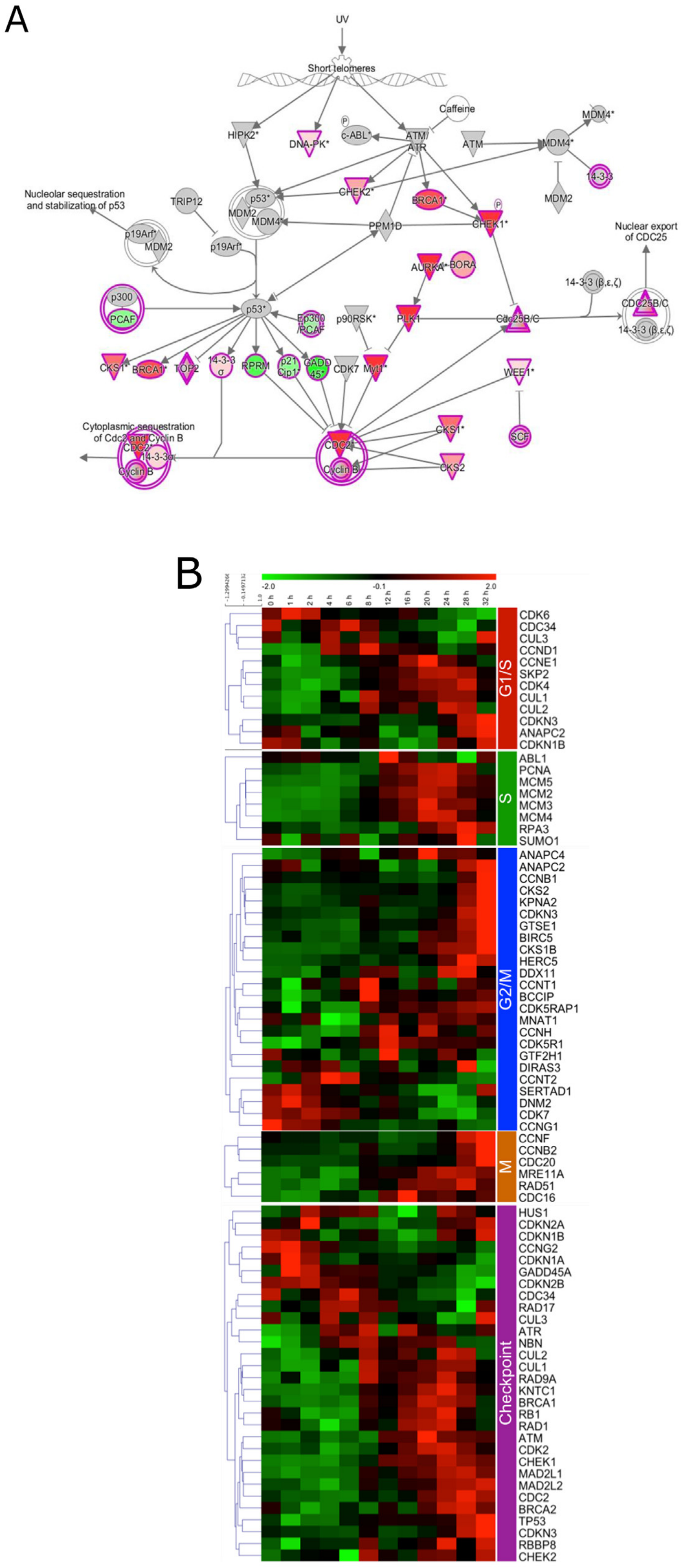

Supplementary Figure 4: Estrogen treatment alters expression of cell cycle genes in an estrogen-responsive cell line. **(A)** Gene expression network array of MCF-7 cells treated for 48 hours with DMSO or estrogen shows up-regulation of G2/M-phase genes with estrogen treatment: up-regulated (red/pink), down-regulated (green) and unchanged genes (grey). **(B)** *In silico* analysis of cell cycle genes based on published datasets of MCF-7 cells treated with estrogen in a time-dependent manner (0-32 hours). The analysis indicates that estrogen drives cell cycle progression and this can be seen mainly in checkpoint and G2/M-phase genes.

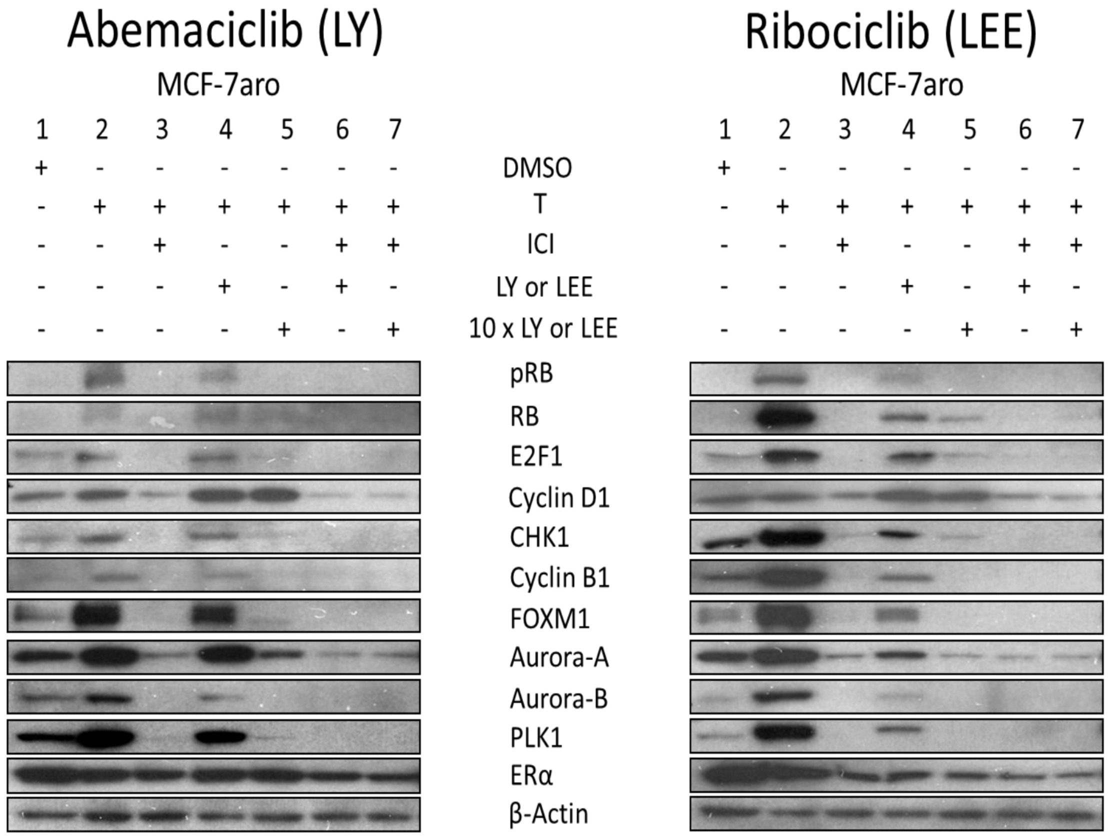

Supplementary Figure 5: Abemaciclib (LY) or Ribociclib (LEE) and ICI exhibit synergism in HR+/endocrine therapy responsive cell line. Western blot analysis of MCF-7aro cell line treated 48 hours with abemaciclib (LY) or ribociclib (LEE) shows reduction of pRB/RB and G2/M-phase protein levels. Combination with ICI treatment exhibits significant cell cycle protein reduction versus single treatment. Concentrations of inhibitors used were the IC50 values.

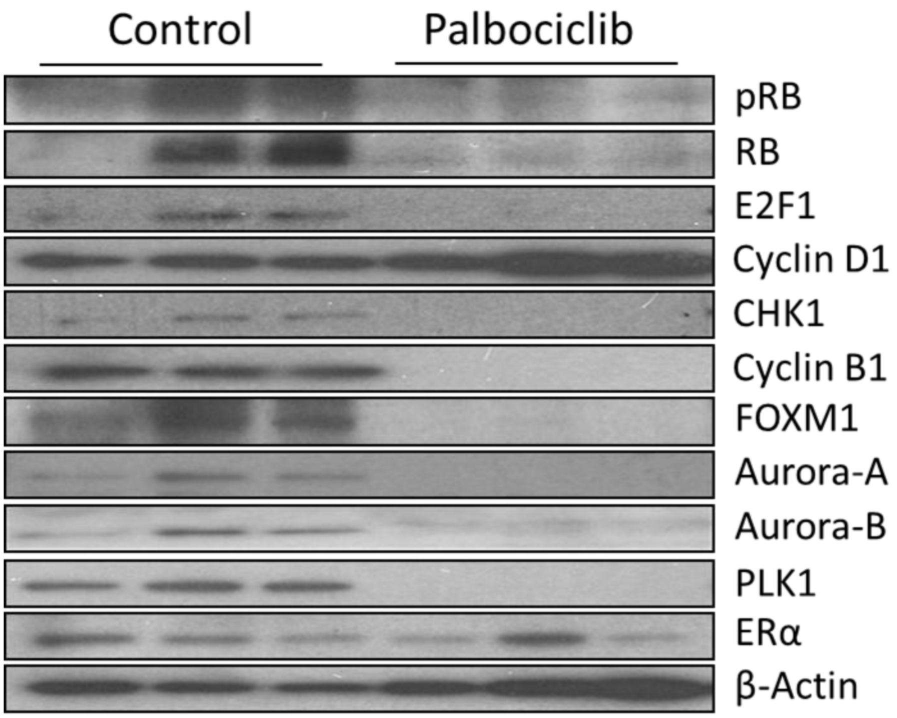

Supplementary Figure 6: Palbociclib treatment targets cell cycle networks in a PDX tumor model. PDX GS4 (ER+/PR+/HER2-) tumor model treated for 3 days with palbociclib shows treatment reduced expression of G1/S- and G2/M-phase proteins.

Supplementary Table 1: MCF-7aro cell cycle analysis

Treatment	G1	S	G2
DMSO	93.0%	2.2%	3.5%
T/DMSO	66.5%	17.2%	14.9%
T/ICI	94.1%	0.8%	4.1%
T/PD	79.7%	9.5%	9.3%
T/PD/ICI	93.7%	0.7%	4.3%

Supplementary Table 2: Common genes affected by estrogen treatment.

See Supplementary File 1
