# Supplementary material for: ERα-mediated cell cycle progression is an important requisite for CDK4/6 inhibitor response in HR+ breast cancer
Source: Oncotarget. 2018 Jun 12;9(45):27736–51. doi: 10.18632/oncotarget.25552 (PMC6021239; doi:10.18632/oncotarget.25552)
Supplement: Supplementary file 2 [file oncotarget-09-27736-s002.docx]

**Supplementary Table 2: Common genes affected by estrogen treatment**

|  | **C4-12** | | | **MCF-7** | | |  | | | |
| --- | --- | --- | --- | --- | --- | --- | --- | --- | --- | --- |
| **Symbol** | **Fold Change** | **p.value** | **Status** | **Fold Change** | **p.value** | **Status** | **Overall Status** | | | **Estrogen Regulated** |
| B3GNT6 | 3.1 | 2.39E-18 | E2 Up | 3.34 | 1.92E-10 | E2 Up | Both E2 Up | Yes | | |
| C3 | 1.56 | 3.57E-07 | E2 Up | 2 | 1.11E-04 | E2 Up | Both E2 Up | | Yes | |
| C5AR2 | 5.31 | 1.36E-28 | E2 Up | 6.15 | 5.44E-10 | E2 Up | Both E2 Up | | Yes | |
| C9orf152 | -1.53 | 5.48E-04 | E2 Down | -2.31 | 3.30E-04 | E2 Down | Both E2 Down | | No | |
| CA12 | 1.52 | 4.46E-19 | E2 Up | 2.22 | 5.33E-03 | E2 Up | Both E2 Up | | Yes | |
| CD22 | 2.27 | 2.02E-13 | E2 Up | 2.19 | 3.41E-03 | E2 Up | Both E2 Up | | Yes | |
| COL6A3 | 1.62 | 9.00E-20 | E2 Up | 1.55 | 1.72E-05 | E2 Up | Both E2 Up | | No | |
| CXCL8 | -1.58 | 8.58E-04 | E2 Down | -2.31 | 9.05E-04 | E2 Down | Both E2 Down | | No | |
| GAS6-AS2 | 1.71 | 7.01E-05 | E2 Up | 2.07 | 2.41E-03 | E2 Up | Both E2 Up | | Yes | |
| GREB1 | 1.84 | 2.22E-42 | E2 Up | 3.16 | 5.85E-05 | E2 Up | Both E2 Up | | Yes | |
| GRIK3 | 1.61 | 1.97E-13 | E2 Up | 2.83 | 7.91E-06 | E2 Up | Both E2 Up | | Yes | |
| HR | 1.67 | 3.05E-09 | E2 Up | 3.14 | 4.54E-05 | E2 Up | Both E2 Up | | No | |
| HSPB8 | 2.6 | 3.73E-83 | E2 Up | 4.17 | 7.32E-07 | E2 Up | Both E2 Up | | Yes | |
| IFIT1 | -1.57 | 1.42E-23 | E2 Down | -2.55 | 3.37E-04 | E2 Down | Both E2 Down | | No | |
| IGFBP4 | 1.89 | 5.17E-27 | E2 Up | 4.23 | 5.78E-07 | E2 Up | Both E2 Up | | Yes | |
| KRT13 | 3.53 | 7.58E-19 | E2 Up | 2 | 1.62E-03 | E2 Up | Both E2 Up | | Yes | |
| OAS2 | -1.66 | 6.73E-11 | E2 Down | -2.51 | 1.14E-04 | E2 Down | Both E2 Down | | No | |
| PGLYRP2 | 2.25 | 2.41E-10 | E2 Up | 5.03 | 9.69E-09 | E2 Up | Both E2 Up | | Yes | |
| PGR | 1.82 | 2.70E-40 | E2 Up | 23.92 | 1.99E-25 | E2 Up | Both E2 Up | | Yes | |
| RNF223 | 4.26 | 6.08E-17 | E2 Up | 2.97 | 1.60E-04 | E2 Up | Both E2 Up | | No | |
| RSAD2 | -2.2 | 9.47E-30 | E2 Down | -2.19 | 1.85E-03 | E2 Down | Both E2 Down | | No | |
| SYNDIG1 | 1.51 | 7.01E-07 | E2 Up | 2.64 | 9.39E-06 | E2 Up | Both E2 Up | | No | |
| SYTL5 | 3.39 | 2.82E-89 | E2 Up | 2.58 | 6.75E-04 | E2 Up | Both E2 Up | | Yes | |
| TFF1 | 3.58 | 2.41E-06 | E2 Up | 5.21 | 1.50E-08 | E2 Up | Both E2 Up | | Yes | |
| TH | 1.89 | 1.45E-05 | E2 Up | 2.23 | 3.38E-03 | E2 Up | Both E2 Up | | Yes | |
| TSKU | 1.72 | 1.57E-32 | E2 Up | 2.99 | 1.34E-04 | E2 Up | Both E2 Up | | Yes | |
| TUBA3D | 2.35 | 5.08E-11 | E2 Up | 2.6 | 8.71E-04 | E2 Up | Both E2 Up | | Yes | |
| TUBA3E | 4.96 | 4.88E-26 | E2 Up | 4.76 | 1.15E-09 | E2 Up | Both E2 Up | | Yes | |
| WNT16 | 7.67 | 2.43E-165 | E2 Up | 3.46 | 8.17E-08 | E2 Up | Both E2 Up | | Yes | |
